# Toward the Existence of a Sympathetic Neuroplasticity Adaptive Mechanism Influencing the Immune Response. A Hypothetical View—Part I

**DOI:** 10.3389/fendo.2019.00632

**Published:** 2019-09-20

**Authors:** Emanuel Bottasso

**Affiliations:** Department of Pathology and Physiology, Faculty of Medicine, Centro de Altos Estudios en Ciencias Humanas y de la Salud, Universidad Abierta Interamericana, Rosario, Argentina

**Keywords:** neuro-immune interaction, sympathetic fibers, secondary lymphoid organs, neural plasticity, semaphorins, neurotrophins

## Abstract

The nervous system exerts a profound influence on the function of the immune system (IS), mainly through the sympathetic arm of the autonomic nervous system. In fact, the sympathetic nervous system richly innervates secondary lymphoid organs (SLOs) such as the spleen and lymph nodes. For decades, different research groups working in the field have consistently reported changes in the sympathetic innervation of the SLOs during the activation of the IS, which are characterized by a decreased noradrenergic activity and retraction of these fibers. Most of these groups interpreted these changes as a pathological phenomenon, referred to as “damage” or “injury” of the noradrenergic fibers. Some of them postulated that this “injury” was probably due to toxic effects of released endogenous mediators. Others, working on animal models of chronic stimulation of the IS, linked it to the very chronic nature of processes. Unlike these views, this first part of the present work reviews evidence which supports the hypothesis of a specific adaptive mechanism of neural plasticity from sympathetic fibers innervating SLOs, encompassing structural and functional changes of noradrenergic nerves. This plasticity mechanism would involve segmental retraction and degeneration of these fibers during the activation of the IS with subsequent regeneration once the steady state is recovered. The candidate molecules likely to mediate this phenomenon are also here introduced. The second part will extend this view as to the potential changes in sympathetic innervation likely to occur in inflamed non-lymphoid peripheral tissues and its possible immunological implications.

## Introduction

The nervous (NS) and immune (IS) systems have aroused increasing interest in biomedical research during the last 50 years accompanied by important advances in the understanding of their functioning. Since the 1970s many research groups have attempted to understand the complex relationship between both systems and how the NS modulates the immune response, considering that the IS works in a physiological framework instead of being a self-regulated system. The two major pathways involved in such neuroendocrine-immune interactions are the hypothalamic-pituitary-adrenal (HPA) axis and the autonomic nervous system (ANS), mainly the sympathetic nervous system -SNS- [reviewed in (1, 2)]. However, the proper mechanisms mediating this crosstalk are only partly understood. The present review will provide some clues compatible with the existence of a still hypothetical neural plasticity mechanism arising during the development of an immune response in secondary lymphoid organs (SLOs), probably influential in immunological terms.

### Sympathetic Innervation of SLOs

In 1984 Felten et al. (3) made a detailed description of the sympathetic innervation of mouse lymph nodes by means of fluorescent histochemistry. Noradrenergic fibers enter the hilus and distribute either into a subcapsular nerve plexus or travel via blood vessels through the medullary cords. These fibers go along with small vessels into the parenchyma of paracortical region (the T zone, where the antigenic presentation takes place) and cortical region (the B zone), surrounding the lymphoid follicles. Individual lymph nodes receive their sympathetic input from postganglionic neurons depending on the region where this lymphoid organ is located (4).

After these initial studies, noradrenergic innervation in the rat spleen was identified by the same group by using immunohistochemistry for tyrosine-β-hydroxylase -TβH- (the rate-limiting enzyme of catecholamine biosynthesis and specific marker for sympathetic fibers) and electron microscopy (5–7). Noradrenergic fibers enter the spleen around the splenic artery, travel with the vasculature in plexuses, and continue along the trabeculae in trabecular plexuses. Fibers from both the vascular and trabecular plexuses go into the white pulp and continue along the central artery and its branches. Noradrenergic varicosities radiate from these plexuses into the T zone, that is, the periarterial lymphatic sheath (PALS). The B zone is also innervated, and the red pulp contains scattered fibers, primarily associated with the plexuses along trabeculae and surrounding tissues. The well-characterized co-transmission phenomenon of sympathetic fibers, assures the release of neuropeptide Y (NPY) and adenosine triphosphate (ATP) together with adrenaline and noradrenaline (NA) by the sympathetic terminals and varicosities (8), for which NPY positive fibers can also be identified in the rat spleen (9). In addition, the prevertebral sympathetic ganglia associated with the celiac-mesenteric plexus were found to provide the major sympathetic input to the spleen (10). A similar noradrenergic innervation pattern has recently been demonstrated in the human spleen (11).

NA released from the sympathetic nerves mediate its effects by primarily interacting with the α- and β-adrenergic receptors (12, 13) expressed on immune cells (4). Furthermore, immune cells also express functional purinergic (14–16) and NPY-Y receptors (17, 18), allowing ATP and NPY to respectively interact with them. A non-synaptic neurotransmission has been suggested at this level, so the released neurotransmitters may act in a paracrine fashion (19), with no synaptic neuro-immune communication being demonstrated so far (20).

### Changes in Noradrenergic Innervation of SLOs During the Immune Response

Upon activation, the IS elicits a rapid and selective increase in splenic sympathetic activity in the early phase of the immune response (21–24). Conversely, many studies seem to indicate that once the IS becomes fully activated, such sympathetic activity significantly decreases in SLOs. Pioneering work from Besedovsky and del Rey's group (25, 26) described a very important decrease in NA content in lymphoid organs like the spleen and lymph nodes during the IS activation. They initially showed that 3 days after challenging rats with a harmless antigen such as sheep red blood cells -the timepoint when the IS begins to get fully activated- there was a substantial decline of NA in SLOs. Following that, it was observed that specific-pathogen-free rats, which usually have contact with environmental antigens and therefore possess a stimulated IS, had lower concentrations of NA within their spleens, compared to germ-free rats from the same strain. Importantly, this decrease in NA was observed regardless of the results were expressed as NA content per gram tissue or per total spleen. There were no changes in NA content from non-lymphoid organs.

Expanding these studies (27), the same group also reported a positive correlation between the magnitude of the immune response and the decrease of splenic NA. Investigations on whether the development of sympathetic innervation in the spleen was affected by lymphoid cells (28), revealed no difference in NA content in the spleen from newborn athymic nude mice and normal thymus-bearing littermates, but demonstrated higher NA levels in 7-, 11-, and 21-day old athymic mice. Remarkably, the reconstitution of newborn nude mice by thymus transplantation or thymocyte injection resulted in splenic NA levels comparable to those seen in normal mice. Fluorescence histochemistry for the visualization of splenic sympathetic fibers showed a higher number of fluorescent fibers and enhanced fluorescence intensity within the spleens from 21-day old athymic mice, compared to normal counterparts or thymus-grafted mice.

Following these demonstrations, other studies consistently observed a decreased sympathetic activity in different animal models, both during acute and chronic immune responses. For instance, decreased levels of NA were observed in the spleens of mice challenged with staphylococcal enterotoxin B superantigen (29). In the same way, MRL-lpr/lpr male and female mice -lacking functional Fas expression and prone to develop lymphoproliferative autoimmune diseases- presented decreased levels of splenic NA prior to the onset of splenomegaly (30–32). Decreased levels of splenic NA were also observed in acute *Trypanosoma cruzi*-infected mice (33), in a murine AIDS model induced by the LP-BM5 mixture of murine retroviruses (34), and in Lewis rats with adjuvant-induced arthritis (35). More recently, a marked loss of sympathetic noradrenergic nerves in patients who died from sepsis has also been shown (11). In fact, spleens from half of septic patients lacked noradrenergic fibers whereas presence of these nerves from the septic group was significantly reduced as compared to control samples obtained from patients with no inflammatory diseases.

Some of these studies also assessed the presence of noradrenergic fibers, by fluorescence histochemistry for catecholamines (28, 30), immunohistochemistry for TβH (11, 32–34) or both (35). In all cases, there was a consistently marked decrease in sympathetic fibers in the non-vascular areas within the spleen during the IS activation. In some studies fibers were only present around vascular structures whereas in other samples only very rare positive fibers were found. Interestingly, one of these studies (34) assessed total nerve fibers density through immunohistochemistry for protein gene product 9.5 (PGP 9.5), a constitutive protein found in the cytoplasm of all central and peripheral neurons, whose presence is independent of neural activity. The PGP 9.5 staining pattern resulted to be very similar to the TβH one, that is, a much less PGP 9.5 staining in spleens from mice in the acute phase of the viral infection, compared to control spleens. Also, PGP 9.5 positive nerve fibers were rarely found once the IS was already activated.

Most researchers regarded this decreased sympathetic activity as a pathological process, referred to as an “injury,” “damage,” or even “destruction” of sympathetic fibers within the spleen during immune activation. In this sense, different hypotheses were postulated concerning possible toxic effects of the high levels of NA released at the very onset of the response, prior to full activation of the IS, or oxidative stress caused by the immune response itself. The use of models of chronic inflammation led other research groups to propose that the phenomenon was due to the chronicity of the process. In this regard, Besedovsky and del Rey interpreted this observation, from the very beginning, as a part of a physiological mechanism, by which the “*decrease in noradrenergic activity late during the immune response*” was “*a way of releasing immune cells from the inhibitory effects of NA, favoring the take-off of the adaptive response*” (36).

**Figure 1:**
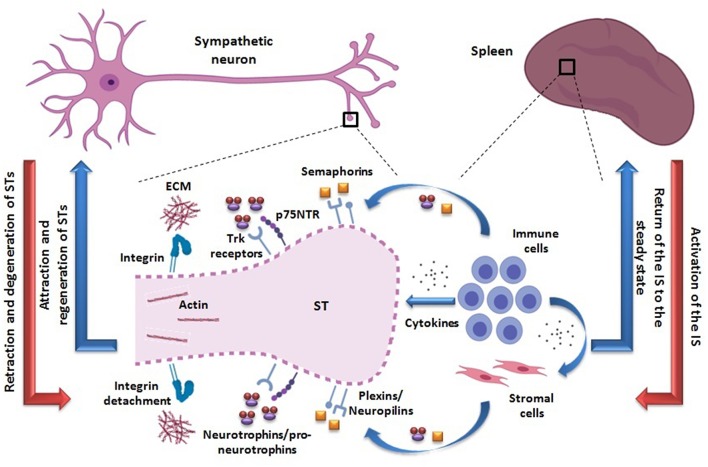
Proposed adaptive mechanism of neural plasticity from STs innervating secondary lymphoid organs such as the spleen. The activation of the IS may be accompanied by retraction and axonal degeneration of STs (red arrows). The activated immune cells are able to produce semaphorins and pro-neurotrophins/neurotrophins, binding to their receptors -plexins/neuropilins and Trk/p75NTR, respectively- probably expressed on STs. In particular, p75NTR may be re-expressed in a pro-inflammatory milieu. The action of these molecules may lead to an inhibition of STs integrin-mediated adhesion to ECM and depolymerization of their actin cytoskeleton, thus favoring their retraction and axonal degeneration (dotted line). In addition, semaphorins and neurotrophins/pro-neurotrophins may be produced by other non-lymphoid cell types as well (i.e., stromal cells), under cytokine influence. A direct action of cytokines as playing a role on STs retraction cannot be disregarded. Once the immune response ceases, the IS returns to the steady state and STs may regenerate, recovering the usual splenic innervation pattern (blue arrows). Neural and immunological phenomena are summarized on the left and right sides, respectively. STs, sympathetic terminals; IS, immune system; Trk, tropomyosin-related kinase; p75NTR, p75 neurotrophin receptor; ECM, extracellular matrix.

Based on currently available evidence, the existence of an adaptive mechanism of neural plasticity involving sympathetic terminals retraction during IS activation within SLOs can be envisioned (Figure 1). In fact, activated immune cells can produce different molecules likely to mediate axonal retraction and/or segmental axonal degeneration of sympathetic fibers innervating SLOs. On the other hand, in an inflammatory milieu, cytokines can also stimulate the production of these same molecules by other non-immune cells. Within this hypothetical neural plasticity adaptive mechanism that may modify the way by which the NS modulate the IS functioning, two major compounds are quite likely to play a role, semaphorins and neurotrophins (see Box 1 and Table 1).

Box 1 **Semaphorins and their receptors**

Semaphorins are a family of soluble, transmembrane or cell-anchored proteins that contain a common “sema” domain of about 400 amino acids [reviewed in (37–39)]. They were initially described as guiding molecules during the development of the NS, with capacity to attract or repel axonal growth cones so that they could reach the appropriate targets. They have been grouped into 8 classes based on their structural characteristics. Semaphorins of class 1 and 2 belong to invertebrates, those of class 3–7 to vertebrates and those of class 8 are coded by viruses. There are also subclasses that are designated with letters (for example, Sema3A or Sema4D) and currently there are more than 30 different types of semaphorins. Class 1, 4–6 semaphorins are transmembrane proteins, class 7 members are glycosylphosphatidylinositol-linked, while class 2, 3 and viral semaphorins are soluble proteins. Class 4, 5, and 7 semaphorins can be cleaved and released extracellularly.

Semaphorins bind receptors expressed in neurons called plexins. Plexins are grouped into four classes called A–D. Four types of plexin A, three types of plexin B, one plexin C, and one plexin D have been described. There are also two types of plexins in invertebrates. In contrast to the remaining classes, class 3 semaphorins require a co-receptor binding to signal through class A plexins (40). These co-receptors are transmembrane proteins called neuropilins. They have a short intracellular domain devoid of intrinsic catalytic activity and functioning as a ligand-binding partner in co-receptor complexes for both plexins and vascular endothelial growth factor receptors.

Beyond their role in the development of the NS (37–39, 41), these proteins continued to be expressed in the adult brain, where they are linked to processes such as the modulation of synaptic activity in the hippocampus (42). As well as participating in the modulation of intrinsic NS functions, two elegant studies (43, 44) demonstrated that the 65KDa isoform of Semaphorin 3A (produced by endothelial cells of the medial eminence of the hypothalamus) and Semaphorin 7A produced by tanycytes (in both cases under steroid stimuli) can act on Neuropilin-1 or on Plexin C1. This is followed by an attraction or retraction, respectively, of the nerve terminals of the gonadotropin releasing hormone neurons, thus favoring the release of the neurohormone toward the anterior pituitary in different phases of the ovarian cycle.

Remarkably, it has also been shown that these proteins play a role in processes unrelated to axon guidance or to synaptic plasticity, such as organogenesis, vascularization, angiogenesis, neuronal apoptosis and tumor progression (45, 46). Finally, it has been demonstrated that both semaphorins and plexins are expressed in immune cells, where they interact and exert influences on functions as diverse and critical as cell-to-cell contact, modulation of immunological synapses, regulation of immune cell activation (by serving as costimulatory molecules), proliferation, differentiation, cell migration or production of cytokines [(47, 48), reviewed in (49–51)]. For instance, a transmembrane semaphorin, 4D Semaphorin, is weakly expressed on T cells, B cells and antigen presenting cells such as dendritic cells (DCs). Its expression increases radically after activation with different immunological stimuli and in these circumstances, 4D Semaphorin suffers a proteolytic cleavage resulting a soluble form of it. 6D Semaphorin is present in T cells, B cells and NK cells, whereas 7D Semaphorin is found in activated T cells and in double positive thymocytes. Sema4A is expressed on antigen-presenting cells and Sema4C is upregulated on follicular T helper cells (52, 53). Regarding plexins, Plexin-A1 is highly expressed by mature DCs, while Plexin-A4 is located in T cells, DCs, and macrophages. Plexin-B1 is expressed on activated T cells and follicular dendritic cells. As to Plexin-B2, its expression was found in macrophages, DCs, and plasmacytoid dendritic cells, being also highly expressed by germinal center B cells (53). Plexin-D1 is present in CD4^+^ CD8^+^ double positive thymocytes (49, 51).

Current data on soluble semaphorins, which can be released into the extracellular medium and exert their action in the vicinity without any cell-to-cell contact, is perhaps even more intriguing for the purposes of this viewpoint. For instance, 3A Semaphorin is produced by activated T cells and one of its functions would be to inhibit the proliferation of T cells themselves and the production of cytokines (54). The same study shows that CD4^+^ T cells express higher levels of 3A Semaphorin than CD8^+^ T cells. Expression of 3A Semaphorin has also been observed in human blood peripheral monocytes, further increasing when monocytes are differentiated with macrophage colony-stimulating factor under conditions that promote a macrophage M2 phenotype (alternatively activated macrophages). DCs also express 3A Semaphorin and their maturation induced by both TNF-α and IL-1β or by CD40L significantly increases the expression of Sema3A mRNA (55). More recently, T-cell precursors in the thymus of humans were also found to express another soluble semaphorin, 3F Semaphorin (56).

**Neurotrophins and their receptors**

Since the discovery of nerve growth factor (NGF) by Rita Levi Montalcini (57), great advances have been made in the field of neurotrophins. These molecules, defined primarily as neural survival stimulants during development in sympathetic neurons, constitute a group of soluble proteins produced by many different cell types. They are called NGF, brain derived neurotrophin factor (BDNF), neurotrophin 3 (NT-3) and neurotrophin 4 (NT-4) [reviewed in (58–61)], acting on transmembrane receptors expressed primarily on neural cells: the tropomyosin-related kinase (Trk) receptors and p75 neurotrophin receptor (p75NTR). NGF binds preferably to TrkA, BDNF and NT-4 to TrkB and NT-3 to TrkC. All neurotrophins can interact with p75NTR. The interactions of neurotrophins with Trk receptors are of high affinity, whereas the binding of neurotrophins to p75NTR has a very low affinity. However, the binding of NGF to TrkA and that of BDNF to TrkB are of low affinity. Furthermore, p75NTR can act as a co-receptor by increasing the affinity of neurotrophins for Trk receptors.

The signaling systems of these receptors, mainly those of the p75NTR, are very complex and some controversies exist in this regard (62–65). Acting on Trk receptors, the neurotrophins promote cell survival and growth, mostly by stimulating the activation of PI-3 kinase-AKT and Ras-ERK pathways. On the other hand, p75NTR, a member of the tumor necrosis factor (TNF) receptor superfamily, lacks intrinsic catalytic activity and signals through a series of interactions with different proteins through its intracellular juxtamembrane and death domains. This receptor contributes to cell survival or to neurite outgrowth (by activating or regulating the NF-κB pathway and Rho activity, respectively), as well as cell migration. Nevertheless, when Trk receptors activation is reduced or absent, high levels of p75NTR expression can mediate axonal degeneration or induce apoptosis through increased ceramide production and activation of c-Jun N-terminal kinase, caspases, and p53 (66–68).

Since the binding affinity of neurotrophins to p75NTR is very low compared to their affinity for Trk receptors, the view that neurotrophins were just cell survival promotors prevailed for a long time. However, it had been proven that during development NGF and BDNF exerted functionally antagonistic actions on sympathetic neuron growth and target innervation, acting via TrkA or p75NTR to promote or inhibit growth, respectively (69). Even so, the high-affinity *bona fide* ligands of p75NTR that may mediate cell death remained largely elusive. Finally, in 2001 Lee et al. (70), showed that pro-NGF was a high-affinity ligand for p75NTR. It was also demonstrated that pro-NGF induces p75NTR-dependent apoptosis in cultured sympathetic neurons with minimal activation of TrkA-mediated differentiation or survival. Like many other proteins, neurotrophins are synthesized as proforms that are further cleaved intracellularly by furin or other proconvertases to release their mature form. Pro-neurotrophins had always been considered biologically inactive precursors until the work by Lee et al. (70) showed that neurotrophins may be released as pro-neurotrophins into the extracellular medium and then undergo extracellular cleavage by extracellular proteases such as serine protease plasmin and selective matrix metalloproteinases. Thus, the biological action of neurotrophins is regulated by proteolytic cleavage, with proforms preferentially activating p75NTR to mediate apoptosis or axonal degeneration and mature forms conversely promoting survival, axonal and dendritic growth, via Trk receptors (71).

Neurotrophins and pro-neurotrophins not only act during development but also in the adult brain, for instance, mediating synaptic plasticity. Within the hippocampus, mature BDNF facilitates long-term potentiation through TrkB, whereas long-term depression is facilitated by pro-BDNF through p75NTR activation (72–74). At the peripheral level the application of pro-BDNF induces a dramatic decrease in synaptic efficacy in the neuro-muscular plaque followed by a retraction of the presynaptic terminals, this effect being mediated by p75NTR (75). This lend support to the view that post-synaptic secretion of pro-BDNF may stabilize or cause retraction of the presynaptic terminal depending on the proteolytic conversion of this molecule to its mature form, or not.

On the other hand, vascular endothelial cells are able to synthesize BDNF (76), whereas platelets store and release it upon activation, predominately through proteinase-activated receptor-1 stimulation by thrombin, and plasmin, among other mediators (77, 78). Hemostasis and the IS are linked in different physiological and pathological conditions, mainly via the complement system. Then, some authors have proposed that these interactions are particularly relevant in adaptive and maladaptive neural plasticity within the central nervous system, at the level of the neurovascular unit (the blood-brain barrier on the one side, and neurons, glia, and extracellular matrix on the other side). Such interactions are thought to be quite influential in the development of different conditions, like Alzheimer's disease, neuro-inflammation, stroke, neoplastic, and psychiatric disorders (79–81).

Beyond these considerations, it is important to highlight the well-recognized expression of neurotrophins in different inflammatory milieu and unstimulated immune cells, further increasing upon their activation (82–95). This will constitute a central issue as to the view proposed in the present work.

**Table 1:** Summary of different types of semaphorins and neurotrophins, along with their receptors, actions and presence in immune cells.

**Molecules**	**Types**	**Receptors on neural cells**	**Activities**	**Expression on immune cells**
Semaphorins	Class 1 and 2 in invertebrates; class 3, 4, 5, 6, and 7 in vertebrates; class 8 in viruses Subclasses designated with letters (i.e., Sema3A or Sema4D, etc.) Class 1 and 4–7 semaphorins are transmembrane proteins; class 2, 3 and viral semaphorins are soluble proteins Sema4D has a soluble form	Plexins: grouped into four classes (A–D), and presenting many different subtypes Neuropilins: co-receptors for class 3 semaphorins	Axon guidance molecules with capacity to attract or repel axonal growth cones Modulation of synaptic activity in the hippocampus Plasticity in uterine sympathetic nerves Modulation of hormone release in the pituitary Organogenesis, angiogenesis, neuronal apoptosis and tumor progression Many immune functions: cell-to-cell contact, modulation of immunological synapses, regulation of immune cell activation (by serving as costimulatory molecules), proliferation, differentiation, cell migration, and cytokine production	4A Semaphorin: expressed on antigen-presenting cells (52) 4C Semaphorin: upregulated on follicular T helper cells (53) 4D Semaphorin: expressed on T cells, B cells and dendritic cells (DCs), markedly increased upon activation [reviewed in (49–51)] 6D Semaphorin: present in T cells, B cells and NK cells (49–51) 7D Semaphorin: seen in activated T cells and in double positive thymocytes (49–51) 3A Semaphorin: produced by activated CD4^+^ and CD8^+^ T cells, human blood peripheral monocytes, macrophages and DCs (54, 55) 3F Semaphorin: present in T-cell precursors in the human thymus (56)
Neurotrophins (all of them are soluble proteins)	Nerve growth factor (NGF) Brain derived neurotrophin factor (BDNF) Neurotrophin 3 (NT-3) Neurotrophin 4 (NT-4) Pro-forms and mature forms are released. Pro-forms can be cleaved intra or extracellularly	Tropomyosin-related kinase (Trk) receptors A, B and C, high-affinity receptors for mature forms of neurotrophins p75 neurotrophin receptor (p75NTR), “low-affinity receptor,” showing high affinity for pro-forms p75NTR is specifically re-expressed under cytokine influence and during injury [reviewed in (96–104)]	Mature forms promote cell survival, axonal and dendritic outgrowth, mainly via Trk receptors Neurotrophins (mainly their pro-forms) can also mediate axonal degeneration or apoptosis via p75NTR, when Trk receptors activation is reduced or absent Synaptic plasticity within the hippocampus Plasticity in uterine sympathetic nerves Immune functions: modulation of immune cells apoptosis, proliferation and cytokine production	BDNF: *in vitro* on activated human T cells, B cells and monocytes (82) NT-3, BDNF, TrkB, and TrkC: human immune cells (83) NGF, BDNF, NT-3, and NT-4/5: rat T cells, significantly increased upon antigen activation (84) BDNF, pro-BDNF, and Trk receptors: human B cells (92) TrkA: human monocytes (95) p75NTR: murine and human plasmacytoid dendritic cells (94) p75NTR and pro-BDNF: murine innate immune cells (93) Elevated levels of neurotrophins found in many different inflammatory scenarios (85–91)

*These molecules are widely expressed in many different cell types, in some cases under the influence of cytokines in an inflammatory milieu*.

### On the Potential Role of Semaphorins and Neurotrophins

There is reason to believe that semaphorins and their receptors may be involved in potential changes in the innervation of SLOs, i.e., in the spleen, during activation of the IS leading to a retraction in sympathetic fibers. Plexin A3 and Plexin A4 (receptors for 3A and 3F Semaphorins) are expressed in sympathetic fibers together with Neuropilin-1 and Neuropilin-2, which collectively are essential for the migration of sympathetic neurons during the development of the ANS (105–107). *In vitro* experiments showed that both Sema3A and Sema3F can repel dissociated neurons from wild-type sympathetic superior cervical ganglia (108). Apparently, Plexin-A3 would be preferentially used in Sema3F/Neuropilin-2 signaling while Plexin-A4 primarily signals downstream of Sema3A/Neuropilin-1. Production of Semaphorin 3A during activation of the IS (54) opens the possibility that this molecule could interact with its canonical receptors Plexin A4 and Neuropilin 1, conceivable expressed in the sympathetic fibers innervating the SLOs, and hence mediating their retraction. On the other hand, in an inflammatory milieu, semaphorins may not only be produced by the immune cells themselves but by other cell types under the influence of cytokines, as reported in other experimental settings (109). Moreover, it cannot be excluded that cytokines induce the expression of other types of plexins or neuropilins on the sympathetic fibers on which other types of semaphorins may act. Alternatively, semaphorins produced during an activation of the IS may bind receptors different from the canonical ones on sympathetic nerves, like the soluble form of 4D semaphorin interacting with CD72 (110).

The proper mechanism by which semaphorins exert their effects has not been fully clarified. It was first described that by binding to plexins (using neuropilins as co-receptors for type 3 semaphorins), semaphorins induce a dramatic depolymerization of the actin cytoskeleton, which normally forms lamellipodia and filipodia (111, 112). A depolymerization of the fascin-associated actin bundles may afford the substrate for actomyosin contractions, thus mediating retraction. In addition, plexin activation leads to a fast disassembling of integrin-based focal adhesive structures, preventing cell adhesion to components of the extracellular matrix (113). Thus, the neurite's entire structure collapses and retracts. The integrity of the actin cytoskeleton is not only essential for the axonal growth cone, but also indispensable at the synaptic terminal level for the maintenance and regulation of vesicles pools, their attachment to the active zone and their exocytosis, thus allowing neurotransmitters release (114). Hence, it may be speculated that semaphorins effects on sympathetic terminals within SLOs not only led to their retraction and collapse but may also elicit a rapid interruption of the release of different neurotransmitters.

Finally, the semaphorins and plexins/neuropilins system can mediate both the retraction and the attraction of nerves. It has already been mentioned the case of 3A and 7A Semaphorins acting on Neuropilin-1 or on Plexin C1 from hypothalamic gonadotropin releasing hormone neurons (43, 44). Moreover, the repulsion elicited by 3D Semaphorin on growth cones of cultured *Xenopus* spinal neurons can be transformed into attraction upon pharmacological activation of the guanosine 3′, 5′-monophosphate (cGMP) and adenosine 3′, 5′-monophosphate (cAMP) signaling pathways (115). In the same way, studies in *Caenorhabditis elegans* with lowered levels of specific RAC GTPases revealed a conversion of cell movement responses to Semaphorin-1 and Plexin-1 from attraction to repulsion (116). In another interesting study, it was shown that Sema5A displays both attractive and inhibitory guidance activities on the development of *fasciculus retroflexus*, a diencephalon fiber tract from rat brains (117). Apparently, the type-1 thrombospondin repeats domain of this semaphorin can mediate regulatory functional interactions with different components of the extracellular matrix determining how Sema5A affects neuronal growth cones. On the other hand, in the zebrafish brain Sema3D seems to conduct axons from the nucleus of the medial longitudinal fasciculus by repulsion, acting through receptors containing Neuropilin-1A (118). Unlike this, the same semaphorin seems to attract telencephalic neurons that form the anterior commissure via receptors containing Neuropilin-1A and Neuropilin-2B. Thus, axons may respond differentially to a single semaphorin, depending on their neuropilin composition. Hence, several signals involving semaphorin and plexin/neuropilin interactions may regulate actin polymerization and depolymerization causing attraction (cytoskeletal growth) or repulsion (cytoskeletal collapse), respectively. In this scenario, it may be assumed that once the immune response is terminated, different signals may concur to mediate sympathetic fibers attraction, thus favoring the reconstitution of the innervation pattern from the steady state. These may include changes in the cytokine environment, changes in the type of semaphorins produced by the immune cells themselves or by other cells under the influence of cytokines, or changes in the type of receptors expressed on sympathetic nerves.

However, regardless of their repelling or attracting action on nerve fibers, semaphorins would not be mediating the physical “disappearance” of sympathetic nerves by axonal degeneration which seems to occur within SLOs during an IS activation (11, 28, 30, 32–35). Therefore, other molecules may be acting concomitantly (Figure 1).

In this regard, neurotrophins and pro-neurotrophins and their receptors are likely to mediate a transient and segmental axonal degeneration of sympathetic nerves -followed by regeneration once the IS returns to a resting state. Importantly, the expression of neurotrophins in lymphocytes and other immune cells in basal conditions, along with a remarkable increase in their production after their activation is well-recognized. In 1999, activated human T cells, B cells and monocytes were shown to secrete bioactive BDNF *in vitro* (82). The same group demonstrated that in T cell lines specific for myelin autoantigens, such as myelin basic protein or myelin oligodendrocyte glycoprotein, BDNF production increased upon antigen stimulation. Moreover, BDNF immunoreactivity was also identified in inflammatory infiltrates in brain from patients with acute disseminated encephalitis and multiple sclerosis. By the same time, another group observed that human immune cells also produced NT-3 mRNA, secreted BDNF, and expressed their specific receptors TrkB and TrkC (83). The Th1 cytokine IL-2 stimulated the expression of TrkB mRNA but not of TrkC, whereas the Th2 cytokine IL-4 enhanced NT-3 but not BDNF mRNA expression. Following that, it was shown that rat T cells expressed NGF, BDNF, NT-3, and NT-4/5 and that the secretion of neurotrophins by T cells was significantly increased by antigen activation (84). By then the dual role of neurotrophins and pro-neurotrophins acting on Trk receptors and p75NTR facilitating antagonistic processes was unknown (70, 71), therefore there was no interest in assessing whether immune cells released mature forms of neurotrophins or pro-neurotrophins. Moreover, since neurotrophins were thought to stimulate only neuronal survival and most of these experiments were conducted in animal models of central nervous system (CNS) inflammatory diseases, inflammatory infiltrates in conditions like multiple sclerosis were supposed to exert a “neuroprotective” role (119, 120).

The relationship between neurotrophins and immune-mediated phenomena was initially envisioned by Aloe et al. (85) and Bracci-Laudiero et al. (86) who reported elevated levels of NGF in the synovial fluid of patients with chronic arthritis and in sera from patients with systemic lupus erythematosus (SLE), in the latter case positively correlated with disease severity. More recently, it was also demonstrated that circulating levels of NGF and BDNF are increased in SLE patients, with severe lupus flares showing augmented NT-3 levels (87). Other studies also demonstrate increased values of neurotrophins in the cerebrospinal fluid of children with viral meningoencephalitis (88), in plasma from rheumatoid arthritis patients (89) as well as in many other inflammatory and autoimmune states [reviewed in (90)], including the bronchoalveolar lavage fluid of patients with pulmonary sarcoidosis (91). In this study, immunohistochemistry revealed the expression of NGF, BDNF and NT-3 in sarcoid granulomas. Again, no characterization on whether it corresponded to pro-forms or the mature ones was attempted.

Further work led to envisage different immunological functions for neurotrophins. Apparently, they would act primarily in autocrine loops on immune cells by virtue of the expression of both neurotrophins and Trk receptors on these cells. In this sense, B lymphocytes have been shown to produce and release both the mature form of BDNF and pro-BDNF to the extracellular medium that may modulate apoptosis on the same B cells (92). On the other hand, neurotrophin signaling has also been shown to regulate immune cell proliferation and cytokine secretion, via the p75NTR and TrkA receptors (93–95).

Independently of the type of neurotrophins produced and released by immune cells during activation, analyzing the expression of their receptors in neural cells in an inflammatory context is critical to support the above-stated neural plasticity hypothesis. As regards p75NTR, this receptor is widely expressed in the developing NS, including sympathetic neurons, while most cells no longer express it at adult stages. Surprisingly, many different types of injury and cellular stressors are potent inducers of p75NTR re-expression in neuronal and glial cells [reviewed in (121)]. For instance, p75NTR is up-regulated in rat dorsal root ganglion neurons after peripheral nerve transection (96), in corticospinal neurons after axotomy in mice (97), in rat retina following ischemic injury (98), in ischemic stroke in rat striatal interneurons (99), in hippocampal neurons during rodent seizures (100), in spinal cord motor neurons in murine and human amyotrophic lateral sclerosis (101), as well as in glial cells in multiple sclerosis plaques (102) and in basal forebrain neurons of patients with Alzheimer's disease (103). Interestingly, inflammatory cytokines such as IL-1β and TNF-α have been shown to up-regulate p75NTR in neurons and glial cells of the CNS (104). In this study IL-1β induced p75NTR expression via p38 MAPK in hippocampal neurons, and via p38 MAPK and NF-kB in astrocytes, whereas TNF-α induced p75NTR expression via NF-kB both in hippocampal neurons and in astrocytes. Hence, a pro-inflammatory cytokine milieu, common to some of the pathological conditions reproduced in these models of neural injury, may regulate the re-expression of p75NTR on neural cells. Whether sympathetic fibers innervating SLOs react by up-regulating p75NTR upon cytokine signals during an immune response remains and intriguing possibility.

It is worth noting that, p75NTR mediated axonal degeneration should not always be regarded as a pathological process, but as a physiological mechanism, in some cases. *In vitro* models of sympathetic axon competition revealed how winning axons release BDNF, further interacting with p75NTR on losing axons to promote their degeneration. This mechanism is essential for the normal development of neuronal circuits (122, 123). Moreover, axonal degeneration takes place in the intact rodent adult brain via a p75NTR and myelin-dependent mechanism (124), thus precluding septal cholinergic axons (where the expression of p75NTR is maintained in adult life), from an abnormal growth onto myelinated tracts, in the *corpus callosum*. In the same vein, transient loss of sympathetic nerves in SLOs should not be necessarily regarded as harmful but as part of a neural plasticity adaptive mechanism (Figure 1).

Accordingly, the pro-inflammatory cytokine environment present during the IS activation may lead to the re-expression of p75NTR in the sympathetic nerves innervating the SLOs, with neurotrophins produced by lymphocytes mediating a segmental and transient physiological axonal degeneration of those fibers, and hence explaining some of the former observations (11, 28, 30, 32–35). On the other hand, released cytokines may also induce the expression of neurotrophins in other non-immune cell types, further contributing to this mechanism (Figure 1). For instance, IL-1β, IFN-γ, and IL-4 were found to regulate NGF and BDNF expression in human culture bronchial smooth muscle cells (125) whereas NGF was significantly increased in keratinocytes during different skin inflammatory diseases, with TNF-α probably mediating this effect (126–129). As seen with semaphorins eliciting both attraction and retraction of nerve fibers, neurotrophins may promote antagonistic effects being able to mediate either degeneration or axonal growth. In this way the restoration of the innervation pattern of SLOs once the immune response is over, may be explained by modifications in different signals (i.e., changes in the cytokine environment leading to possible alterations in the expression of Trk receptors and p75NTR in sympathetic nerves, changes in the production of neurotrophins and pro-neurotrophins, or changes in factors favoring their intracellular or extracellular cleavage).

### Sensitive and Parasympathetic Innervation of SLOs

Concerning sensory spleen innervation, the presence and distribution of positive fibers for substance P -SP-, one of the main neurotransmitters of sensitive fibers, was described in rats (130). In this study, SP positive nerve fibers entered the spleen with the splenic artery in the hilar region, arborized along the venous sinuses, and extended from these larger plexuses into trabeculae and the surrounding red pulp. In the white pulp, SP positive nerve fibers were found in the marginal zone, and in the outer regions of the PALS among T lymphocytes. SP positive nerve fibers were observed in association with the splenic capsule, the central arteries of the white pulp, or the follicles. However, retrograde tracing studies were unable to find the neuronal cell bodies of such fibers in dorsal root ganglia or nodose ganglia in the rat (10). So, no definitive evidence was found for the sensory input to the spleen and according to the more influential authors in this issue, sensory neuropeptide-positive fibers identified in the spleen would not be involved in providing sensory feedback from this immune organ (4).

Although there are only a few studies on this subject, one report did obtain neuroanatomical evidence in the sense that lymph nodes may receive a sensory afferent supply (131) because retrograde tracing studies in the tracheobronchial lymph nodes of guinea pigs identified neurons in cervical dorsal root ganglia. According to some authors this may have a functional sense, since unlike the spleen, lymph nodes play fundamental roles in local immune responses of the organism (4). On the other hand, careful search of the literature revealed no studies as to possible changes in the sensitive innervation of lymph nodes, and density, quantity or distribution of SP positive fibers within the spleen during an immune activation.

In relation to cholinergic fibers, neuroanatomical evidence for a parasympathetic input to the lymph nodes or to the spleen is lacking (4, 132, 133). Nevertheless, a particular subset of splenic memory T cells has been shown to produce acetylcholine (ACh), able to elicit anti-inflammatory responses via α7 nicotinic ACh receptors on macrophages (134). Apparently, vagal nerve stimulation may result in splenic sympathetic release of NA, stimulating β2-adrenergic receptors on cholinergic T cells and thus provoking ACh release (134, 135).

## An Integrative View

The adaptive branch of the IS has the impressive capacity to produce a specific response against a given non-self-antigen upon its encounter. This response involves antigen recognition, the activation and clonal expansion of specific lymphocytes, followed by the production of cytokines or other molecular mediators, the synthesis of specific antibodies, and migration of immune cells to specific sites; collectively implying a huge energetic and metabolic cost. Provided the response eliminates the triggering insult, the entire storm ceases and the system recovers its steady state.

As stated, many observations from the last four decades have shown that, after an initial increase in sympathetic activity at the very onset of the immune response (21–24), a significant decrease of noradrenergic transmission within the SLOs occurs once the IS becomes activated (11, 25–35). Expanding the view of a physiological mechanism (36), it can be now hypothesized that such phenomena reflect the existence of an adaptive neural plasticity program of the sympathetic fibers innervating SLOs. Thus, retraction and segmental and transient axonal degeneration would occur during an ongoing immune response, with further regeneration once the response achieves its goal and the system returns to a resting state (Figure 1).

Axonal degeneration does occur in a physiological manner, as it was found in certain areas of the brain during development and adult life (122–124). P75NTR up-regulation was also induced by IL-1β and TNF-α, two of the major pro-inflammatory cytokines released during immune activation (104). Moreover, in different neural stress/injury situations and inflammatory scenarios, a re-expression of p75NTR was observed in diverse types of neurons and glial cells (96–103). Considering current knowledge about the p75NTR-mediated effects, many neuroscientists have wondered why the system would respond to neural injury by up-regulating a receptor capable to mediate axonal degeneration and even neuronal apoptosis. While seeming initially unsound, Ibáñez et al. (121) suggested that injury and inflammation induction of p75NTR in cells that expressed the receptor earlier in development may mirror the existence of neural plasticity programs in those situations that to some extend recapitulate a developmental mechanism. Whether sympathetic nerves innervating SLOs react by up-regulating p75NTR during an activation of the IS remains to be established. If so, the existence of a neural plasticity program working in this context would be strengthened.

Although neurotransmitters of the ANS released from the axonal terminals and varicosities are thought to act on the immune cells in a paracrine fashion [non-synaptic neurotransmission (19)], it can be speculated that the neuro-immune interface within the SLOs constitutes a sort of synapse. Therefore, the immune post-synaptic component may generate positive and negative signals that further modify, retrogradely, the structure and function of the pre-synaptic component. The existence of such post-synaptic signals has long been described, like those from the neuro-muscular plaque (136–138).

It is now clear that the NS possesses a remarkable and unexpected plasticity, allowing to modulate critical physiological functions. As above-mentioned, steroid-stimulated endothelial cells from the hypothalamic medial eminence and tanycytes produce and release different semaphorins. These molecules cause attraction or retraction of the nerve terminals of the gonadotropin releasing hormone neurons, regulating the release of the neurohormone toward the anterior pituitary in different phases of the ovarian cycle (43, 44). Another impressive example of neural plasticity, of currently unknown significance, occurs in the uterus, in which sympathetic axons degenerate or regenerate depending on whether estrogen levels rise or decline, respectively (139–147). This phenomenon was found to be mediated by different molecules produced by the myometrium under estrogen's influence, including neurotrophins and pro-neurotrophins acting on Trk receptors and p75NTR, as well as semaphorins.

The above-mentioned evidence suggests that semaphorins and neurotrophins or pro-neurotrophins, and their respective receptors, are quite likely candidates to mediate this hypothetical neural plasticity phenomenon of sympathetic fibers innervating SLOs. These molecules, essential during the development of the NS, continue to be expressed in adult life, fulfilling fundamental and very different functions. For instance, the expression of transmembrane and soluble semaphorins and neurotrophins by activated immune cells and in different inflammatory settings was widely documented (47–56, 82–95), as did their production by non-immune cell types under the influence of cytokines in an inflammatory milieu (109, 125–129). As stated, by acting on their receptors expressed in axons, they can mediate both the collapse, retraction and degeneration of neural processes as well as their attraction and regeneration (70, 71, 115–118). Changes either in the type of molecules produced by immune cells or other cell types or in the receptor expressed in the neural processes on which they act, along with modifications in the local cytokine environment may explain such antagonistic actions. Far from exerting their actions separately, neurotrophins and semaphorins were shown to activate cellular pathways that can interact downstream (148–150). Accordingly, neurotrophins may be able, in some cases, to quickly modulate the response of a given axon to semaphorins, so that the final response would be dynamic, relying on the interaction of cytoplasmic signals elicited concurrently by both types of molecules.

The possible participation of molecules other than semaphorins and neurotrophins in this mechanism cannot be excluded. There is evidence for inflammatory cytokines to exert both positive and negative direct regulatory roles in neurogenesis, neural stem cell proliferation, fate specification, young neuron migration and neuronal maturation (151) as well as synaptic plasticity (152). On the other hand, as happened with semaphorins, netrin-1, a protein formerly described as an axon guidance molecule (153), seems to be involved in multiple physiological and pathological conditions, such as organogenesis (154), angiogenesis (155), tumorigenesis (156), and inflammation (157, 158). Netrin-1 is a bifunctional axonal guidance cue, capable of attracting or repelling developing axons via activation of different receptors (159). Interestingly, it has been shown that netrin-1 is essential for the development of arterial sympathetic innervation in mice. Netrin-1 is produced by arterial smooth muscle cells and arterial innervation required its interaction with one of its receptors on sympathetic growth cones (160). A participation of this molecule in the proposed mechanism is therefore possible.

Also, the existence of signals from de CNS contributing to this hypothetical mechanism should be considered. Very early, it was observed that the immune response elicits the activation of different areas of the CNS, such as the hypothalamus (161). In these circumstances the activation of the HPA and gonadal axes is a well-known physiological response (162, 163). Moreover, the activation of other centers within the cortico-limbic region is involved in the so-called sickness behavior (164). Hence, as different areas of the CNS become activated during an ongoing immune response, central responses may be elaborated in turn, thus affecting autonomic activity in the periphery and probably contributing to the decreased sympathetic activity during immune activation.

Much research is needed to properly demonstrate the existence of this putative neural plasticity mechanism and the processes accounting for it. Even when the intimate machinery mediating changes in sympathetic innervation in SLOs were unveiled, too many questions would remain unanswered. Evidence for changes in lymph nodes innervation is less abundant than that for the spleen. This may be due to the technical advantages of this latter organ and to the characteristics of the animal models used in these studies. Since the lymph nodes apparently do possess sensory innervation, it would also be interesting to know what happens to SP positive fibers during an immune activation.

Another essential question is whether changes in the sympathetic, sensitive and/or parasympathetic innervation within peripheral non-lymphoid tissues accompany the development of an inflammatory process, together with the recruitment of activated immune cells at the site of phlogosis. If molecules proposed to mediate changes in local innervation -semaphorins and neurotrophins- are released by activated immune cells or other cytokine-stimulated cell types, this may be the case. If so, the emerging question deals with the potential clinical consequences of this, considering the ANS regulates many biological functions.

Perhaps the most intriguing matter is what would be the physiological and immunological goal of this hypothetical neural plasticity mechanism. Considering the accumulated evidence on the broad relationship between the NS and the IS (1, 2), this neural plasticity mechanism may ensure a differential neural modulation of the IS in its diverse functional-associated activation states. It was stated that decreased noradrenergic activity during an ongoing immune response is “*a way of releasing immune cells from the inhibitory effects of NA*” for a proper adaptive response to be developed (36). This interpretation is in line with the β-adrenergic-receptor-mediated immunosuppressive effects of NA on such cells, particularly on Th1-type responses (1, 2, 165–167); the most consistent view on the immunomodulating effect of the ANS. Even if this turns out to be true, current evidence also supports a proinflammatory role of the SNS. In fact, SNS signaling has also been shown to play an important role in the induction of proinflammatory cytokines (168, 169), as well as in contributing to proliferation and mobilization of myeloid lineage immune cells in the first steps of inflammation [reviewed in (170)]. Moreover, two different groups have reported that peripheral sympathectomy in mice led to a significant increase in CD4^+^Foxp3^+^ Treg compartment in SLOs (171, 172). Consequently, the physiological meaning of this neural plasticity mechanism may be even more complex. Some of these issues will be better addressed in the second part of this work.

## Author Contributions

The author confirms being the sole contributor of this work and has approved it for publication.

### Conflict of Interest Statement

The author declares that the research was conducted in the absence of any commercial or financial relationships that could be construed as a potential conflict of interest.
